# Knowledge, Attitude, and Confidence Related to Antibiotic Prescribing and Antimicrobial Resistance Among Undergraduate Students at a Dental School in South Africa

**DOI:** 10.7759/cureus.112949

**Published:** 2026-07-19

**Authors:** Suwayda Ahmed, Rukshana Ahmed, Renier Coetzee, Razia Zulfikar Adam

**Affiliations:** 1 Dentistry, University of the Western Cape, Cape Town, ZAF; 2 School of Public Health, University of the Western Cape, Cape Town, ZAF

**Keywords:** antibiotic prescribing, antimicrobial resistance, antimicrobial stewardship, dentistry, south africa, undergraduate dental students

## Abstract

Introduction

Antibiotic prescribing has seen a consistent rise in recent years, with dentists emerging as significant contributors to systemic antibiotic prescriptions. Decisions regarding antibiotic prescriptions are guided by clinical indications, patients’ oral health status and medical history. Although undergraduate dental education highlights the treatment of most oral infections by clinical intervention, evidence exists of increased antibiotic prescribing among dentists. This highlights the need for antimicrobial resistance (AMR) awareness and antimicrobial stewardship in undergraduate dental education.

Aim

To determine the knowledge, attitudes, and confidence related to antibiotic prescribing and AMR awareness among undergraduate dental students.

Methods

A cross-sectional, questionnaire-based study employing convenience sampling was conducted among undergraduate students at a dental school in South Africa. Responses were compiled in a Google spreadsheet (Google LLC, Mountain View, CA, USA) and exported to IBM SPSS Statistics for Windows, Version 30 (Released 2024; IBM Corp., Armonk, New York, United States) for descriptive, chi-square/Freeman-Halton exact, and Kruskal-Wallis H analyses.

Results

A total of 173 of 240 eligible students participated (n=173, 72% response rate). Self-reported knowledge improved with year level, demonstrating a statistically significant association between year of study and knowledge ratings (χ²=50.17, p<0.001). Knowledge improved with progression through the programme and was not uniformly distributed among the year groups. Students demonstrated low confidence when determining the appropriate antibiotic dose and interval (n=116, 67.1% low confidence) as well as appropriate treatment duration (n=111, 64.2% low confidence).

Conclusion

This study demonstrates varying levels of knowledge, attitudes, and confidence in relation to antibiotic prescribing and AMR awareness. This underscores the need to strengthen dental school curricula to ensure undergraduate dental students practice appropriate antibiotic prescribing and antimicrobial stewardship.

## Introduction

Antimicrobial resistance (AMR) has been recognized as a public health crisis, the impact of which is felt worldwide. The rise in AMR rates has been the focus of global health research, public awareness efforts and health workers’ education [1,2].

Regardless of the increased effort to combat increasing AMR rates, evidence suggests that AMR continues to be one of the most threatening public health concerns, with a projected 10 million global deaths per year forecast by 2050 [1-4]. South Africa is not immune to the threat of rising AMR rates, with 9500 deaths linked to AMR in 2019. The reporting of AMR rates in South Africa is often insufficient due to several factors, including limited resources, inadequate microbiological laboratory testing, and poor infrastructure. As a result, the true burden of AMR in South Africa may be higher than currently reported [5].

In light of this global public health crisis, AMR has the potential to undermine our ability to effectively treat common diseases, as the prevalence of AMR continues to increase [1]. Various factors contribute to rising AMR rates, of which the overuse and misuse of antibiotics are the most dominant [6,7]. Various studies have identified dentists as one of the leading prescribers of systemic antibiotics [8-10]. In clinical dentistry, antibiotics are only indicated in the event of systemic involvement or during treatment measures in which the patient’s immune system is unable to fight infection [11-14].

This emphasizes the importance of a strong foundation of knowledge necessary to make an accurate diagnosis and assess the need for antibiotic prescription among dentists and undergraduate dental students. Through the inclusion of responsible antibiotic prescribing principles, AMR awareness, and antimicrobial stewardship (AMS) principles as essential components of the dental curriculum, a greater understanding and increased awareness can be cultivated, with the potential for transformation within the dental profession [2,12]. Previous studies have demonstrated varying degrees of knowledge, attitudes, and confidence regarding antibiotic prescribing, as well as varying levels of AMR awareness among undergraduate dental students [15-17].

A study led by Mubarak et al. (2024) showed that dental students prescribed antibiotics inappropriately, with notable differences observed between senior and junior students. The researchers emphasized the need for adherence to guidelines and educational initiatives to enhance the quality of dental programmes [15]. Examining students' knowledge and AMR awareness provides insight into what was learned during undergraduate training. This demonstrates the knowledge that will be applied in their professional careers [18]. Although dental students in South Africa prescribe antibiotics only under direct clinical supervision, evidence suggests that prescribing habits and attitudes are formed during undergraduate healthcare training, during contact with clinical supervisors, patients, and country-specific healthcare systems [19,20].

Research suggests that increased levels of knowledge of antibiotic prescribing among undergraduate dental students have a direct correlation with decreased inappropriate antibiotic prescribing practices. This emphasizes the benefit of assessing and improving these concepts during undergraduate training [21]. While previous research studies have observed antibiotic prescribing knowledge among dental students globally, limited research exists in investigating the knowledge, attitudes, and confidence among undergraduate dental students within the South African context [15-17,22,23].

This study aims to investigate the knowledge, attitudes, and confidence of undergraduate dental students relating to antibiotic prescribing knowledge and AMR awareness at a dental school in South Africa. To the best of the researchers’ knowledge, this is the first such study conducted at a dental school in South Africa.

## Materials and methods

Study design

A quantitative cross-sectional study design was conducted to assess the knowledge, attitudes, and confidence in antibiotic prescribing and AMR awareness among undergraduate dental students.

Study setting

The study was conducted at the Faculty of Dentistry, University of the Western Cape (UWC) in South Africa. The Faculty of Dentistry at UWC is the largest among the four dental schools in South Africa and is responsible for training approximately 50% of all new dentists nationally [24].

Sample size

The sample size was calculated at a 95% confidence level and 5% precision for a finite population of N=240, resulting in n=148. All 240 eligible students were invited to participate to maximize the response rate. A total of 173 students responded, resulting in a 72% response rate.

Study duration

The study was conducted from 8 August 2024 to 31 August 2024.

Sampling

A non-probability convenience sampling method was used.

Inclusion criteria

The study included students enrolled in Years 3, 4, and 5 of the Bachelor of Dental Surgery (BDS) program.

Exclusion criteria

Students enrolled in Years 1 and 2 of the BDS program were excluded from the study.

Data collection

A quantitative cross-sectional study design, using a structured questionnaire based on previously validated instruments, was used [23,25]. The questionnaires were distributed to the students in hard-copy format. The completed responses were collected from participants in sealed envelopes and subsequently transferred and recorded on Google Forms (Google LLC, Mountain View, CA, USA) by the primary investigator for data management and analysis. All data was password-protected, and access was restricted to the primary investigator. Participation was voluntary and anonymous, with no identifying information collected. Informed consent was obtained from all participants prior to questionnaire administration. The students were given two weeks to complete the questionnaire, with reminder messages sent after one week to increase the response rate.

A pilot study was conducted with 20 students using convenience sampling, three weeks prior to the main data collection, in order to establish content and face validity. Face validity was established by the pilot participants, who described the questions as clear and appropriate. Content validity was established through expert review by faculty staff. The participants of the pilot study were excluded from the main study. The questionnaire items were analyzed using descriptive statistics, and no scales or composite scores were created; thus, reliability testing was not performed.

The questionnaire comprised 47 multiple-choice and closed-ended questions across three sections (see the Appendices). It collected demographic and educational information, assessed knowledge of antibiotic prescribing, antibiotic prophylaxis, and AMR, and evaluated confidence in antibiotic prescribing. Knowledge was assessed using clinical scenario-based questions requiring participants to indicate whether antibiotics were indicated for common dental conditions. Correct responses to clinical scenarios were benchmarked against established antibiotic prescribing guidelines [26,27]. To maintain a clear distinction between a lack of knowledge and the presence of incorrect knowledge, 'Don't know' responses were categorized as 'knowledge uncertainty' and analysed as a separate category rather than being aggregated with incorrect answers.

Statistical analysis

The responses to the questionnaires were compiled in a Google Sheets spreadsheet (Google LLC), ensuring efficient data aggregation. The dataset was subsequently cleaned, coded, and exported to IBM SPSS Statistics for Windows, Version 30 (Released 2024; IBM Corp., Armonk, New York, United States) for further statistical analysis. Descriptive statistics were used to summarize the demographic information and characteristics of the study participants. Categorical variables, such as gender and study level, were presented as frequencies (n) and percentages (%). To examine the associations between categorical variables, the chi-square or Freeman-Halton extension analyses were employed, where appropriate.

Students' self-rated antibiotic prescribing knowledge was measured using five response categories (Excellent, Above Average, Average, Below Average, and Very Poor). The chi-square test of independence was used to analyze the differences in the distribution of these response categories across the three-year groups. For ordinal data collected using 5-point Likert scales, items such as AMR awareness, knowledge and attitudes toward antibiotic prescribing, and confidence in prescribing antibiotics, the Kruskal-Wallis H test was used to compare differences across the year groups. Statistical significance was set at p < 0.05. Missing data were minimal for most questionnaire items, with missing responses excluded from the final analysis and percentages calculated using the number of valid responses.

## Results

Participant characteristics

A total of 173 students participated in the study, of whom 107 (62%) were female, and 66 (38%) were male. The largest proportion of participants were in their fourth year of study (n=69, 40%) followed by third years (n=63, 36%) and fifth years (n=41, 24%).

Modules influencing prescribing knowledge

Students identified various modules as sources of their antibiotic prescribing knowledge, depending on the year of study. Third-year students acknowledged Dental Pharmacology as the primary course contributing to their knowledge and decision-making skills regarding antibiotics, followed by Maxillofacial and Oral Surgery, Medical Microbiology for Dentistry, and Periodontology. Fourth-year students identified the Maxillofacial and Oral Surgery, Oral Medicine, Anaesthesiology and Sedation, and Paediatric Dentistry modules, while fifth-year students identified the Comprehensive Patient Management module.

Dental students’ antibiotic prescribing knowledge

**Figure 1:**
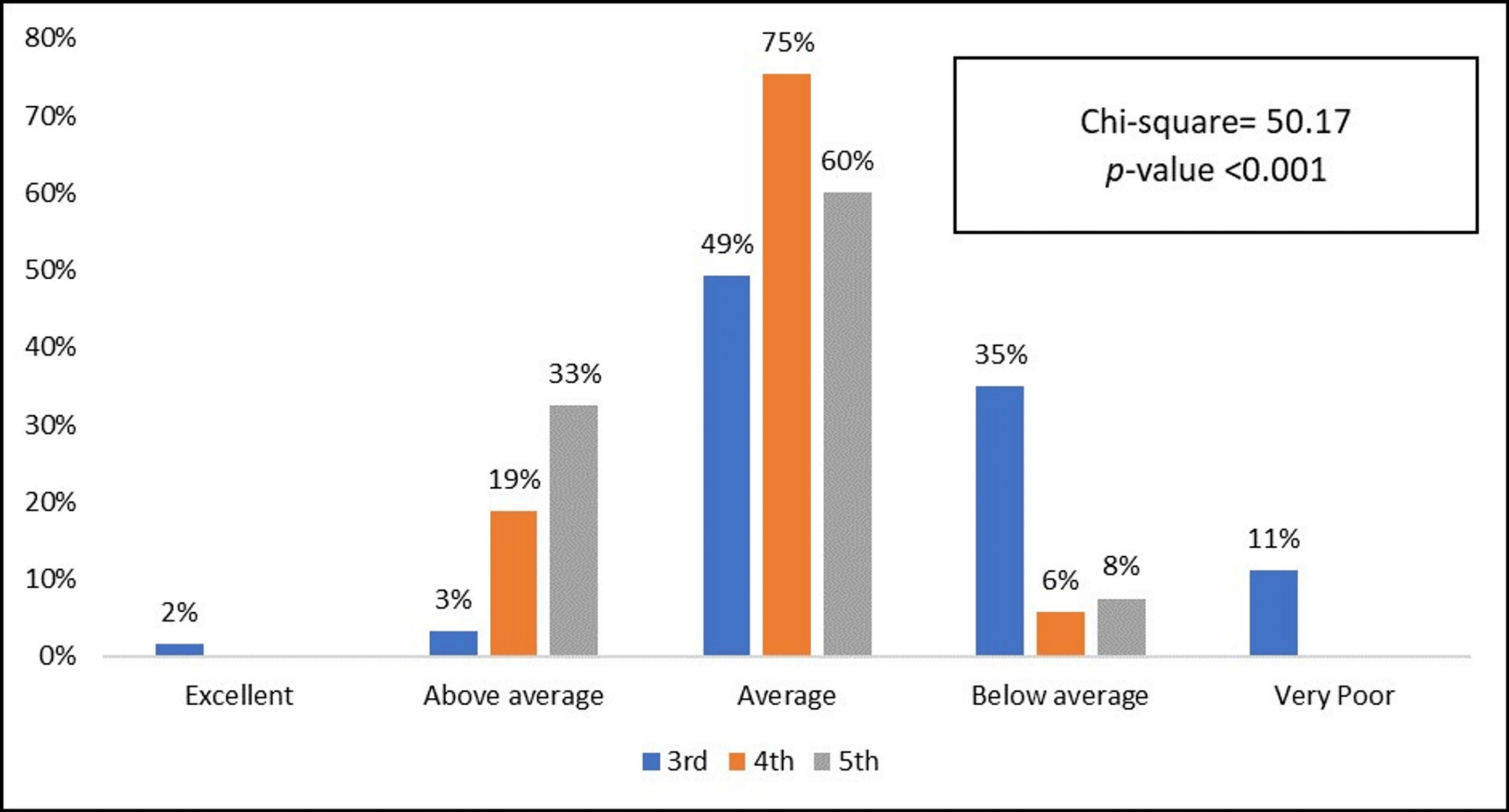
Dental students’ antibiotic prescribing knowledge

A chi-square test of independence was performed to determine whether self-reported knowledge of antibiotic prescribing differed across year groups. Students rated their knowledge using five response categories (Excellent, Above Average, Average, Below Average, Very Poor). The results demonstrated a statistically significant association between year of study and knowledge ratings (χ²=50.17, p<0.001) (see Figure 1). This indicates that students’ self-reported knowledge improved as they progressed through the programme and was not uniformly distributed among the year groups. Third-year students mostly rated their knowledge as 'Average' (n=31, 49%) and 'Below average' (n=22, 35%), and a smaller proportion indicated 'Very poor' (n=7, 11%). Among the fourth-year students, the majority rated their self-reported knowledge as 'Average' (n=52, 75%), followed by 19% (n=13) as 'Above Average'. Among the fifth-year students, 60% (n=25) self-reported their knowledge as 'Average', and a substantial proportion (n=14, 33%) reported their knowledge as 'Above Average'.

Knowledge of antibiotic prescribing across clinical conditions

Chi-square or Freeman-Halton extension analyses demonstrated significant associations between year of study and antibiotic prescribing knowledge for various clinical conditions (Table 1). These findings suggest that the understanding of suitable antibiotic prescribing improves with progression through the undergraduate program, although notable knowledge gaps persist across all year groups.

**Table 1:** Knowledge of antibiotic prescribing for common dental conditions by year of study

Clinical Condition	Third-year students	Fourth-year students	Fifth-year students	Chi-square/Freeman-Halton extension	Cramér’s V
				p-value	
Reversible pulpitis				<0.001	0.375
Don’t know	21 (33.9%)	4 (6.5%)	0 (0.0%)		
No	30 (48.4%)	57 (91.9%)	39 (97.5%)		
Yes	11 (17.7%)	1 (1.6%)	1 (2.5%)		
Irreversible pulpitis				<0.001	0.357
Don’t know	21 (33.9%)	2 (3.2%)	1 (2.5%)		
No	30 (48.4%)	54 (85.7%)	39 (97.5%)		
Yes	11 (17.7%)	7 (11.1%)	0 (0.0%)		
Intraoral draining sinus tract				0.105	0.175
Don’t know	27 (44.3%)	16 (24.2%)	10 (25.0%)		
No	7 (11.5%)	13 (19.7%)	12 (30.0%)		
Yes	27 (44.3%)	37 (56.1%)	18 (45.0%)		
Extraoral draining sinus tract				0.190	0.144
Don’t know	30 (49.2%)	19 (29.7%)	12 (30.0%)		
No	8 (13.1%)	14 (21.9%)	10 (25.0%)		
Yes	23 (37.7%)	31 (48.4%)	18 (45.0%)		
Localized intraoral swelling				<0.001	0.290
Don’t know	21 (34.4%)	11 (16.9%)	2 (5.1%)		
No	10 (16.4%)	24 (36.9%)	25 (64.1%)		
Yes	30 (49.2%)	30 (46.2%)	12 (30.8%)		
Acute facial swelling				0.003	0.232
Don’t know	22 (35.5%)	8 (12.1%)	5 (13.2%)		
No	11 (17.7%)	12 (18.2%)	2 (5.3%)		
Yes	29 (46.8%)	46 (69.7%)	31 (81.6%)		
Dental trauma				0.025	0.183
Don’t know	18 (29.0%)	8 (12.7%)	6 (15.4%)		
No	26 (41.9%)	44 (69.8%)	24 (61.5%)		
Yes	18 (29.0%)	11 (17.5%)	9 (23.1%)		
Periodontal diseases				<0.001	0.332
Don’t know	10 (15.9%)	5 (7.6%)	3 (7.7%)		
No	15 (23.8%)	40 (60.6%)	32 (82.1%)		
Yes	38 (60.3%)	21 (31.8%)	4 (10.3%)		
Pericoronitis				<0.001	0.256
Don’t know	15 (24.2%)	4 (6.3%)	2(5.1%)		
No	8 (12.9%)	17 (27.0%)	18 (46.2%)		
Yes	39 (62.9%)	42 (66.7%)	19 (48.7%)		
Simple extraction				<0.001	0.249
Don’t know	5 (8.1%)	1 (1.6%)	0 (0.0%)		
No	46 (74.2%)	60 (95.2%)	40 (100%)		
Yes	11 (17.7%)	2 (3.2%)	0 (0.0%)		
Surgical extraction				<0.001	0.303
Don’t know	9 (14.5%)	7 (10.9%)	1 (2.6%)		
No	11 (17.7%)	23 (35.9%)	28 (71.8%)		
Yes	42 (67.7%)	34 (53.1%)	10 (25.6%)		
Periapical abscess				<0.001	0.244
Don’t know	4 (6.3%)	3 (4.5%)	1 (2.7%)		
No	1 (1.6%)	6 (9.0%)	11 (29.7%)		
Yes	58 (92.1%)	58 (86.6%)	25 (67.6%)		
Dry socket				<0.001	0.335
Don’t know	12 (19.7%)	5 (8.1%)	3 (7.5%)		
No	14 (23.0%)	39 (62.9%)	32 (80.0%)		
Yes	35 (57.4%)	18 (29.0%)	5 (12.5%)		

For reversible pulpitis, ‘don’t know’ responses decreased from 33.9% (n=21) in the third year to 6.5% (n=4) in the fourth year and 0% (n=0) in the fifth year, while correct responses (‘no’) increased from 48.4% (n=30) to 91.9% (n=57) and 97.5% (n=39) (p-value<0.001; V=0.375). A similar trend was observed for irreversible pulpitis, where ‘don’t know’ responses declined from 33.9% (n=21) to 3.2% (n=2) and 2.5% (n=1), and correct responses (‘no’) increased from 48.4% (n=30) to 85.7% (n=54) and 97.5% (n=39), across the year groups (p-value<0.001; V=0.357). For localized intraoral swelling, uncertainty decreased from 34.4% (n=21) in the third year to 5.1% (n=2) in the fifth year, while correct responses ('no') increased from 16.4% (n=10) to 64.1% (n=25), across the year groups (p-value<0.001; V=0.290). Moderate improvements were observed in pericoronitis, simple extraction, surgical extraction, periapical abscess, and dry socket. For example, in simple extraction, correct responses increased from 74.2% (n=46) in the third year to 100% (n=40) in the fifth year, with ‘don’t know’ responses dropping to 0% (p-value<0.001; V=0.249). In the case of acute facial swelling and dental trauma, statistically significant but smaller differences were observed. In acute facial swelling, ‘yes’ responses increased from 46.8% (n=29) in the third year to 81.6% (n=31) in the fifth year (p-value=0.003; V=0.232). In cases of dental trauma, correct responses (‘no’) increased from 41.9% (n=26) to 61.5% (n=24), while ‘don’t know’ responses decreased modestly (p-value=0.025; V=0.183). In contrast, intraoral and extraoral draining sinus tracts results showed no statistically significant differences across year groups (p-value=0.105 and p-value=0.190, respectively).

Dental students’ antibiotic prescribing attitude

Undergraduate dental students’ attitudes toward antibiotic prescribing varied by year of study (Tables 2-3). Kruskal-Wallis tests demonstrated differences in median scores (1=Strongly Disagree to 5=Strongly Agree) between the year groups when exploring antibiotic prescribing attitudes. Regarding feeling pressured to consider the prescription of antibiotics due to time constraints during dental treatment, third-year students had a median score of 3 (Neutral), while fourth- and fifth-year students both had median scores of 2 (Disagree) (H=17.513, p<0.001). Concerning feeling pressured by patients to prescribe antibiotics, third-year students again showed a median score of 3 (Neutral), compared to median scores of 2 (Disagree) for both fourth- and fifth-year students (H=14.639, p<0.001). These findings suggest that senior students felt less external pressure from patients who requested antibiotics.

**Table 2:** Attitudes towards antibiotic prescribing among undergraduate dental students

	Strongly disagree	Disagree	Neutral	Agree	Strongly agree
I tend to feel more pressured to prescribe antibiotics when there is a shortage of time to complete dental treatment	25 (14.5%)	67 (39.0%)	47 (27.3%)	25 (14.5%)	8 (4.7%)
In certain situations, I feel pressured by patients to prescribe antibiotics	38 (22.1%)	63 (36.6%)	38 (22.1%)	27 (15.7%)	6 (3.5%)

**Table 3:** Association between year of study and attitudes towards antibiotic prescribing

	Third-year students	Fourth-year students	Fifth-year students	Kruskal-Wallis H	p-value
I tend to feel more pressured to prescribe antibiotics when there is a shortage of time to complete dental treatment	3	2	2	17.513	<0.001
In certain situations, I feel pressured by patients to prescribe antibiotics	3	2	2	14.639	<0.001

Dental students’ confidence in antibiotic knowledge and prescribing

Varying levels of confidence were observed across different aspects of antibiotic prescribing (Tables 4-5), with 68 students (39.3%) reporting feeling moderately or very confident when making an accurate diagnosis of infection. In selecting the correct antibiotic, 38.7% (n=67) indicated moderate to high confidence. Managing patients who demand antibiotics showed slightly higher confidence levels, with 53.2% (n=92) of students reporting moderate or high confidence. However, confidence was notably lower when determining the correct antibiotic dose and dosing interval (n=116, 67.1% expressing low to no confidence) and when identifying the appropriate duration of therapy (n=111, 64.2% expressing low to no confidence). These findings highlight key educational gaps and areas where students may benefit from additional support and targeted intervention.

**Table 4:** Dental students ‘confidence in relation to antibiotic knowledge and prescribing

	Not at all confident	Slightly confident	Somewhat confident	Moderately confident	Very confident
Making an accurate diagnosis of infection	16 (9.2%)	38 (22.0%)	51 (29.5%)	60 (34.7%)	8 (4.6%)
Choosing the correct antibiotic	16 (9.2%)	38 (22.0%)	52 (30.1%)	50 (28.9%)	17 (9.8%)
Choosing the correct dose and interval of therapy	34 (19.8%)	30 (17.4%)	52 (30.2%)	47 (27.3%)	9 (5.2%)
Choosing the correct duration of the antibiotic	33 (19.1%)	27 (15.6%)	51 (29.5%)	47 (27.2%)	15 (8.7%)
Handling a patient who demands antibiotics when it is not indicated	26 (15.0%)	27 (15.6%)	28 (16.2%)	41 (23.7%)	51 (29.5%)

**Table 5:** Association between year of study and self-reported confidence in antibiotic prescribing

	Third-year students	Fourth-year students	Fifth-year students	Kruskal-Wallis H	p-value
Making an accurate diagnosis of infection	3	3	4	4.545	<0.001
Choosing the correct antibiotic	3	3	4	4.638	<0.001
Choosing the correct dose and interval of therapy	2	3	3	15.162	<0.001
Choosing the correct duration of the antibiotic	2	3	3	33.509	<0.001
Handling a patient who demands antibiotics when it is not indicated	3	3	4	8.510	<0.001

Awareness of AMR among undergraduate dental students

The majority of students (n=137, 79.1%) demonstrated an understanding of how AMR develops and how it spreads (n=96, 55.5%), as seen in Tables 6-7. A concerning finding was that 46.3% (n=80) of students believed that AMR is primarily a concern within hospital settings. Given that dentists prescribe antibiotics within their scope of practice, primarily as an adjunct to treatment, this perception demonstrates a possible gap in knowledge regarding the role of dentistry with regard to AMR.

**Table 6:** Antimicrobial resistance awareness among undergraduate dental students

Dental students’ awareness of AMR	Strongly disagree	Disagree	Neutral	Agree	Strongly agree
The number of newly discovered antibiotics has increased in recent years.	1 (0.6%)	10 (6.0%)	70 (41.7%)	66 (39.3%)	21 (12.5%)
The phenomenon of antibiotic resistance is mainly a problem in hospital settings	6 (3.6%)	31 (18.3%)	52 (30.8%)	60 (35.5%)	20 (11.8%)
I understand how antibiotic resistance is developed.	1 (0.6%)	7 (4.1%)	25 (14.7%)	98 (57.6%)	39 (22.9%)
I understand how antibiotic resistance is spread	2 (1.2%)	18 (10.7%)	52 (31.0%)	68 (40.5%)	28 (16.7%)

**Table 7:** Association between year of study and antimicrobial resistance awareness

	Third-year students	Fourth-year students	Fifth-year students	Kruskal-Wallis H		p-value
The number of newly discovered antibiotics has increased in recent years	4	3	3	1.294		0.524
The phenomenon of antibiotic resistance is mainly a problem in hospital settings	3	3	3	0.947		0.623
I understand how antibiotic resistance is developed	3	4	4	7.484		0.024
I understand how antibiotic resistance is spread	3	4	3	8.187		0.017

## Discussion

This study offered the baseline evidence on knowledge, attitudes, and confidence of undergraduate dental students concerning antibiotic prescribing and AMR awareness at a dental school in South Africa. Key findings suggest that knowledge gaps are evident across all year groups, although there is some improvement in knowledge with advancing levels of study. This highlights the strengths of the current undergraduate curriculum and suggests areas for focused curriculum enhancement in undergraduate dental education. The results demonstrated a statistically significant association between year of study and knowledge ratings (χ²=50.17, p<0.001), indicating that students’ self-reported knowledge improved as they progressed through the programme and was not uniformly distributed among year groups. It is important to note that the association between self-reported knowledge and year of study is possibly due to the integration of course content and clinical experience. Third-year students would have completed the pharmacology and microbiology modules, but would have had less clinical exposure compared to fourth- and fifth-year students, and would thus lack the clinical context in relation to antibiotic prescribing decisions. Therefore, earlier clinical integration of pharmacology content may strengthen students’ ability to convert theoretical knowledge into clinical practice.

Various studies have demonstrated that the dental profession is at the forefront of inappropriate and overprescribing of antibiotics, which may contribute to the burden of AMR [28,29]. Prescribers of antibiotics have a significant role in reducing AMR onset and spread. It seems reasonable that their knowledge, attitudes, and confidence in antibiotic prescribing, as well as awareness of AMR, are of value in the attempt to manage rising AMR rates [30,31]. The assessment of antibiotic prescribing knowledge, attitudes, and confidence among undergraduate dental students can influence how best to manage the growing threat of AMR and encourage AMS within the dental profession.

Students’ self-reported knowledge of antibiotic prescribing is further expanded in the results of clinical scenarios (Table 1), where notable gaps in knowledge could be observed across all year levels. Most conditions in the oral cavity are of an inflammatory nature, which require clinical operative intervention rather than antibiotic therapy. Oral conditions that require antibiotic therapy are specific to those with associated fever, lymphadenopathy, and trismus [15]. A statistically significant association (p<0.001) between the year groups was observed in the management of certain clinical conditions such as reversible pulpitis, irreversible pulpitis, intraoral swelling, periodontal diseases, pericoronitis, surgical and simple extractions, and periapical abscesses. These findings suggest that greater emphasis on clinical scenario-based exercises may strengthen students' antibiotic prescribing knowledge. Although students may receive theoretical knowledge on antibiotic indication, dosage, and duration, there may not be appropriate consolidation through guided clinical decision-making experiences.

In the current study, students were presented with clinical situations and asked to indicate whether they would prescribe antibiotics or not. When presented with reversible pulpitis, 17.7% of third-year students stated they would prescribe antibiotics, compared to 1.6% in the fourth-year and 2.5% in the fifth-year student groups. The results for antibiotic prescribing for a simple extraction showed that 17.7% of third-year students would prescribe, compared to 3.2% in fourth year and 0% in the fifth year. Additionally, when questioned on antibiotic therapy for a dry socket, 57.4% of third-year students would prescribe antibiotics compared to 29% of fourth-year students and 12.5% of fifth-year students. Similar findings were reported by Salvadori et al. (2019), where 5% of students indicated they would prescribe antibiotics for cases of pulpitis, despite it not being an appropriate indication for antibiotic use [32]. Mubarak et al. (2024) reported that 33.9% of their population sample (which included fourth-, fifth-, and sixth-year students) would consider prescribing antibiotics for a dry socket, and 6% of the sample population would provide an antibiotic prescription for simple extractions, displaying a slight difference when compared to the results of the current study [15].

Variable confidence levels were observed among students in relation to antibiotic prescribing. The results demonstrated moderate confidence in diagnosing infections (39.3%) and selecting appropriate antibiotics (38.7%), but low confidence when determining antibiotic dosing regimens (67.1%) and treatment duration (64.2%). Kruskal-Wallis tests confirmed statistically significant differences in median confidence scores between year groups across all five items assessed (Table 5). Confidence in determining the correct antibiotic dose and interval (H=15.162, p<0.001) and treatment duration (H=33.509, p<0.001) also improved with year of study, from a median of 2 among third-year students to a median of 3 among fourth- and fifth-year students; however, this indicates that uncertainty around the correct dose and duration persists into the later years of the programme and may require targeted intervention.

These findings contrast with the improvement observed in self-reported knowledge results across the year groups (χ²=50.17, p<0.001), which may suggest a disconnect between students' perceived knowledge and their confidence in applying that knowledge in clinical practice. These findings partially align with the study by Bajalan et al. (2022), who also reported moderate confidence ratings among undergraduate dentistry students when it came to prescribing antibiotics; however, their study demonstrated that students rated themselves less confident in relation to selecting an antibiotic regimen to treat infections [23]. The ability to confidently determine when and which antibiotic to prescribe is an integral component of dental education [23]. The increase in knowledge, skills, and confidence of undergraduate students is something to consider in undergraduate clinical courses, as it can influence their clinical practice and competency [33].

Understanding which modules contribute to antibiotic prescribing knowledge is important in order to identify gaps in the curriculum and opportunities for knowledge enhancement. The current study showed that different modules contributed to antibiotic prescribing knowledge at different periods in the undergraduate degree programme. During the third year, the majority of information on antibiotic prescribing and AMR was gained through the Dental Pharmacology and Maxillofacial and Oral Surgery modules. In the fourth year, students acquired this knowledge primarily from the Maxillofacial and Oral Surgery and Oral Medicine modules, while in the fifth year, the Comprehensive Patient Management module was the main contributor. This is comparable to a study by Holz et al. (2021), where Oral Surgery and Pharmacology modules were identified as the primary modules in which antibiotic prescribing curriculum content was focused [17]. A notable gap in antibiotic prescribing knowledge was observed in the Endodontics and Periodontology modules in the fourth year of study. Generally, in endodontic cases, where the patient is immunocompetent, no antibiotic therapy is required. However, in cases of systemic symptoms, immunocompromised patients, and evidence of infection spread, antibiotic prescription may be necessary [34]. Similarly, cases of periodontal infection or periodontal surgery may require adjunctive antibiotic therapy [35]. The results therefore highlight areas where antibiotic prescribing knowledge can be strengthened within these modules.

The overall recognition among students that AMR is a critical issue for the dental profession is an encouraging finding, suggesting that students across all study levels recognize the role of the dental profession in combating rising AMR. Similarly, Ghafoor et al. found that final year students expressed greater awareness of AMR when compared to other year levels [36]. This widespread awareness among future dental prescribers could significantly promote responsible antibiotic prescribing practices.

However, of concern is that 46.3% of students believed that AMR is primarily a hospital-related problem. This misconception underestimates the contribution of dentistry to community prescribing and demonstrates a need for stronger emphasis in undergraduate curricula. Tariq et al. found that most students in their study were aware of AMR, but a major proportion were not aware of the increased mortality rates associated with resistance [37]. AMR contributes to 1.2 million global deaths annually, with an estimated forecast of 10 million deaths by 2050 [38-40]. This highlights the need to emphasize the role that AMR has in mortality rates. Future prescribers are key contributors in managing AMR by reducing antibiotic prescribing and advocating within their roles as healthcare workers.

This current study has some limitations. The participants in this study were recruited using a non-probability convenience sampling method based on accessibility rather than random selection. While this approach was practical for maximizing participation within the study period, it may have introduced selection bias, and the sample may not fully represent all students at the dental school. It is also improbable to completely rule out potential non-responder bias.

As this was a cross-sectional study, causal relationships between year of study and knowledge, attitudes, and confidence cannot be established. The questionnaire items were analyzed using descriptive statistics, and no scales or composite scores were created; thus, reliability testing was not performed, which limits assessment of the internal consistency of the instrument. No adjustment was made for multiple comparisons, which may increase the risk of false-positive findings.

This was a single-site study, limited to a single dental school in the Western Cape; thus, the findings may not be generalizable to other institutions as there may be differences in their undergraduate course, learning outcomes, teaching methods, resources, and other contextual factors, which may influence findings. Quantitative data gained from questionnaires are vulnerable to social desirability bias, due to the subjective nature of self-reporting knowledge, attitudes, and confidence. The participants in this study were undergraduate dental students at a dental school and, as such, they may have over-reported positive attitudes or selected guideline-compliant responses to appear clinically competent, which may not reflect their true knowledge, attitudes, or confidence, or future prescribing behaviour. Nonetheless, it provides baseline data that can assist in addressing any knowledge, attitudes, and confidence limitations.

The inclusion of case-based learning, simulation-based prescribing exercises, and interprofessional education may support the translation of theoretical knowledge to clinical application. The dental profession is multifaceted, and it is therefore important that each discipline within the undergraduate dentistry programme supports the development of a well-structured and current curriculum in relation to antibiotic prescribing, and additionally provides clear guidelines specific to their speciality.

## Conclusions

This study has demonstrated varying levels of knowledge, attitudes, and confidence regarding antibiotic prescribing and awareness of AMR among undergraduate dental students at a South African dental school. Although there is an improvement in antibiotic prescribing knowledge as students reach their senior years, there are still signs of inappropriate prescribing in certain clinical scenarios. Students also demonstrated low confidence in antibiotic prescribing dose and duration across all year groups, which may require specific and targeted curricular intervention. The results reflect the need to further educate undergraduate dental students in relation to judicious antibiotic prescribing, as well as the implications of incorrect and over-prescribing of antibiotics. These findings highlight the need to re-examine the current curriculum and clinical teaching related to antibiotic prescribing and AMR, while also underscoring the importance of aligning the educational content with the established guideline recommendations.

## Appendices

Student questionnaire

Gender

□Male

□Female

□ Do not wish to state

Year of Study

□BDS III

□BDS IV

□BDS V

Section 1: Education

Please tick your appropriate response in the blocks below.

1. Which department/courses contributed to the majority of your knowledge and decision-making skills regarding antibiotics? You may choose more than one.

**Table 8:** BDS III

BDS III	Please tick
Systemic Pathology PAT310	
Principles of Medicine and General Surgery PMG310	
Measuring Health and Diseases MHD320	
Basic Orthodontics ORT320	
Social Sciences and Dentistry SSD320	
Conservative Dentistry CON311	
Maxillofacial and Oral Surgery MFS300	
Medical microbiology for Dentistry MIC355	
Periodontology OMP301	
Dental Pharmacology PCL305	
Dental Prosthetics PRO300	
Radiographic techniques RAT300	

**Table 9:** BDS IV

BDS IV	Please tick
Anaesthesiology and Sedation ANS403	
Conservative Dentistry CON401	
Dental Research DRE411	
Endodontics END400	
Maxillofacial and Oral Surgery MFS400	
Oral Medicine OMP401	
Oral Pathology OPA400	
Orthodontics ORT400	
Paediatric dentistry and techniques PED400	
Periodontology PER400	
Prosthetic Dentistry PRO401	
Diagnostics and Radiology RAD400	
Prevention PRE410	

**Table 10:** BDS V

BDS V	Please tick
Advanced Restorative techniques ART510	
Practice management PRM500	
Comprehensive patient management CPM500	

2. What kind of teaching format has been used during your education on antibiotics? Choose as many as necessary.

**Table 11:** What kind of teaching format has been used during your education on antibiotics? Choose as many as necessary

	Please tick
Lectures	
Case discussions	
E-learning	
Direct Patient care	
Other	

3. Who/what has had the most impact on your decision of when and how to prescribe antibiotics?

Choose as many as necessary.

**Table 12:** Who/what has had the most impact on your decision of when and how to prescribe antibiotics? Choose as many as necessary

	Please tick
Full time faculty in clinic	
Part time faculty in clinic	
Student peers	
Lectures	
Personal experience	
Personal research/investigation	
Other	

Section 2: Knowledge

Please tick your appropriate response in the blocks below.

1. I believe dentistry should play an important role in reducing antimicrobial resistance.

**Table 13:** I believe dentistry should play an important role in reducing antimicrobial resistance

Strongly disagree	Disagree	Neutral	Agree	Strongly agree

2. Do you think that ‘antibiotic resistance’ is an important topic for dentists?

**Table 14:** Do you think that ‘antibiotic resistance’ is an important topic for dentists?

Strongly disagree	Disagree	Neutral	Agree	Strongly agree

3. Are you aware of the guidelines for antibiotic prescribing?

**Table 15:** Are you aware of the guidelines for antibiotic prescribing?

Yes	No

4. Are you aware of guidelines for antibiotic prophylaxis?

**Table 16:** Are you aware of guidelines for antibiotic prophylaxis?

Yes	No

5. Please select the option that best suits your level of agreement with each statement.

Please tick your appropriate response in the blocks below.

**Table 17:** Please select the option that best suits your level of agreement with each statement

	Strongly disagree	Disagree	Neutral	Agree	Strongly agree
In your opinion is the overuse of antibiotics a driver of antibiotic resistance.					
The overuse of antibiotics prescribed by dentists contributes to antibiotic resistance.					
The number of newly discovered antibiotics has increased in recent years					
Vaccination and personal hygiene are important to reduce the number of antibiotics used in the world.					
Self-medication by patients with antibiotics contributes to the increase of antibiotic resistance					
Antibiotic resistance is a significant threat in South Africa.					
To prescribe unnecessary antibiotics is unethical.					
The phenomenon of antibiotic resistance is mainly a problem in hospital settings					
I understand how antibiotic resistance is developed.					
I understand how antibiotic resistance is spread.					
I think the problem of antibiotic resistance will increase over the next years					
I am convinced that new antibiotics will be developed to solve the problem of resistance.					

6. Please answer the following:

I would routinely prescribe antibiotics in the following conditions:

Please tick your appropriate response in the blocks below:

**Table 18:** I would routinely prescribe antibiotics in the following conditions:

Clinical condition for which antibiotics are indicated	Yes	No	Don’t know
Reversible pulpitis			
Irreversible pulpitis			
Intraoral draining sinus tract			
Extraoral draining sinus tract			
Localized intraoral swelling			
Acute facial swelling			
Dental trauma			
Periodontal diseases			
Pericoronitis			
Simple extraction			
Surgical extraction			
Periapical abscess			
Dry socket			

7. In patients with a penicillin allergy which alternative antibiotic do you prescribe? Please choose one.

**Table 19:** In patients with a penicillin allergy which alternative antibiotic do you prescribe?

Clindamycin	
Erythromycin	
Cephalexin	
I don’t know	

Section 3: Confidence

Please tick your appropriate response in the blocks below.

1. I would rate my knowledge about antibiotic prescribing for clinical practice as:

**Table 20:** I would rate my knowledge about antibiotic prescribing for clinical practice as:

Excellent	Above average	Average	Below average	Very Poor

2. How confident do you feel to communicate to patients when antibiotics are not needed?

**Table 21:** How confident do you feel to communicate to patients when antibiotics are not needed?

Not at all	Slightly	Somewhat	Moderately	Very
confident	confident	confident	confident	confident

3. Please select the option that best suits your level of agreement with each statement. Scale from ‘Strongly disagree’ to ‘Strongly agree’.

**Table 22:** Please select the option that best suits your level of agreement with each statement

	Strongly disagree	Disagree	Neutral	Agree	Strongly agree
I tend to feel more pressured to prescribe antibiotics when there is a shortage of time to complete dental treatment					
In certain situations, I feel pressured by patients to prescribe antibiotics					

4. With your current level of understanding how confident would you feel in the following scenarios when prescribing an antibiotic by yourself:

**Table 23:** With your current level of understanding how confident would you feel in the following scenarios when prescribing an antibiotic by yourself:

	Not at all confident	Slightly confident	Somewhat confident	Moderately confident	Very confident
Making an accurate diagnosis of infection					
Choosing the correct antibiotic					
Choosing the correct dose and interval of therapy					
Choosing the correct duration of the antibiotic					
Handling a patient who demands antibiotics when it is not indicated.					

5. With your current level of understanding how confident would you feel in prescribing antibiotic prophylaxis.

**Table 24:** With your current level of understanding how confident would you feel in prescribing antibiotic prophylaxis

	Not at all confident	Slightly confident	Somewhat confident	Moderately confident	Very confident
I can evaluate the necessity of antibiotic use to prevent an infection (antibiotic prophylaxis).					
I can select the most appropriate antibiotic and regimen (dose, intervals, duration) for antibiotic prophylaxis.					
When I am not entirely sure about the necessity of antibiotic prophylaxis, I feel confident in finding the best available clinical practice guideline					
